# Estimating the cost-effectiveness threshold of advanced non-small cell lung cancer in China using mean opportunity cost and contingent valuation method

**DOI:** 10.1186/s12962-023-00487-z

**Published:** 2023-11-02

**Authors:** Qian Peng, Yue Yin, Min Liang, Mingye Zhao, Taihang Shao, Yaqian Tang, Zhiqing Mei, Hao Li, Wenxi Tang

**Affiliations:** 1 Center for Pharmacoeconomics and Outcomes Research, China Pharmaceutical University, Nanjing, China; 2 School of International Pharmaceutical Business, China Pharmaceutical University, Nanjing, China; 3 Department of Public Administration, School of International Pharmaceutical Business, China Pharmaceutical University, Nanjing, China

**Keywords:** QALY, Value, Willingness-to-pay, Cost-effectiveness threshold, Advanced lung cancer, China

## Abstract

**Objectives:**

Monetizing health has sparked controversy and has implications for pricing strategies of emerging health technologies. Medical insurance payers typically set up thresholds for quality-adjusted life years (QALY) gains based on health productivity and budget affordability, but they rarely consider patient willingness-to-pay (WTP). Our study aims to compare Chinese payer threshold and patient WTP toward QALY gain of advanced non-small cell lung cancer (NSCLC) and to inform a potential inclusion of patient WTP under more complex decision-making scenarios.

**Methods:**

A regression model was constructed with cost as the independent variable and QALY as the dependent variable, where the regression coefficients reflect mean opportunity cost, and by transforming these coefficients, the payer threshold can be obtained. Patient WTP was elicited through a contingent valuation method survey. The robustness of the findings was examined through sensitivity analyses of model parameters and patient heterogeneity.

**Results:**

The payer mean threshold in the base-case was estimated at 150,962 yuan (1.86 times per capita GDP, 95% CI 144,041–159,204). The two scenarios analysis generated by different utility inputs yielded thresholds of 112,324 yuan (1.39 times per capita GDP) and 111,824 yuan (1.38 times per capita GDP), respectively. The survey included 85 patients, with a mean WTP of 148,443 yuan (1.83 times per capita GDP, 95% CI 120,994–175,893) and median value was 106,667 yuan (1.32 times the GDP per capita). Due to the substantial degree of dispersion, the median was more representative. The payer threshold was found to have a high probability (98.5%) of falling within the range of 1–2 times per capita GDP, while the robustness of patient WTP was relatively weak.

**Conclusions:**

In China, a country with a copayment system, payer threshold was higher than patient WTP, indicating that medical insurance holds significant decision-making authority, thus temporarily negating the need to consider patient WTP.

**Supplementary Information:**

The online version contains supplementary material available at 10.1186/s12962-023-00487-z.

## Introduction

Although the concept of monetizing health raises ethical concerns, it can serve as an efficient and scientifically informed approach to assist decision-makers in allocating limited resources. For instance, it can aid in the pricing strategies for new health technologies by payers in both public and private sectors. Notably, the National Institute for Health and Care Excellence (NICE) has incorporated value-based pricing into the new Pharmaceutical Price Regulation Scheme (PPRS), and value-based assessment has been integrated into the Technology Appraisal Methods Guide [20]. Moreover, payers in countries such as France, Germany, Sweden, and Canada consider a broader range of value-related attributes in health technology assessments [1, 19].

The most prevalent approach to monetizing health involves estimating payer threshold and patient willingness-to-pay (WTP) for quality-adjusted life year (QALY) gains associated with innovative technologies. However, different stakeholders show distinct preferences towards health gains, leading to variations in threshold and WTP. Medical insurance payers, hereafter referred to as payers, typically establish thresholds based on factors such as health productivity, budget affordability, and even key opinion leaders [3]. In the case of public payers, an accepted and recognized approach involves setting the threshold based on the contribution of health to productivity [38]. A commonly utilized method to measure health productivity is the opportunity cost approach, which quantifies the marginal benefit foregone when replacing existing health technology with new one [18]. Patient WTP has been developed over an extended period and can be assessed through several approaches, including the contingent valuation method (CVM), choice experiments method (CEM), and value of statistical life (VSL). Nevertheless, threshold and WTP from these two stakeholder groups (payers and patients) are infrequently measured or compared within the same population.

In China, there has been a significant increase in the listing of new drugs for the treatment of advanced cancer, with a total of 30 drugs being included in the National Reimbursement Drug List (NRDL) during 2019–2021. The Chinese National Healthcare Security Administration, the largest payer in China, paid $59.356 billion for these drugs in 2018, despite the average price reduction of 56.7% compared to the launch prices and 36% lower than global prices(National Healthcare Security [22, 27]. The Chinese government has recently realized that the universally accepted threshold of 1–3 times per capita GDP per QALY gain, as recommended by the World Health Organization [3], is not financially sustainable for Chinese payer. Local studies have shown that both payer thresholds and patient WTP in the general population are below 1.5 times per capita GDP [6, 16, 28, 40]. However, a single value judgment cannot address all the issues, especially for indications with variable population sizes or more complex payment scenarios. Disease-specific WTP research has been expanding in recent years. Life-saving interventions, such as cancer therapy, are often valued more highly than drugs for common chronic diseases [8, 13, 14, 36], as evidenced by a study reporting a higher cancer patient WTP of $11,301 (4.4 times Vietnam's GDP per capita) [15]. NICE also suggests that payer thresholds for end-of-life treatments can be increased by 70% [4].

This study aims to measure and compare Chinese payer threshold and patient WTP of non-small cell lung cancer (NSCLC), and to provide comprehensive evidence and insights from diverse perspectives, which can inform disease-specific decision-making in a complex and predictable value-based pricing scenario.

## Methods

We selected NSCLC as the target indication due to the extensive availability of data sources, with a total of 17 drugs listed in recent years, and the presence of patient samples with a notably high prevalence. The payer threshold was figured out using the mean opportunity cost method. Specifically, a linear regression model was employed to derive the elasticity coefficient, which was then used to calculate the payer threshold. Patient WTP was assessed through face-to-face interviews using a self-administered questionnaire based on the contingent valuation method (CVM). The study received approval from the Ethics Committee of Jiangsu Cancer Hospital on March 31, 2022 (2022KY-KS017).

### Payer threshold

#### Model assumption

The marginal cost of health produced is an extended measure of traditional opportunity cost that is more stable and easier to calculate [10]. In this study, we refer to the innovative method introduced by Claxton [7], a regression model based on mean opportunity cost was employed, using treatment cost as a proxy variable for cost and QALY as a proxy variable for health benefit. To ensure the model adhered to the assumptions of a linear model, the logarithms of both the independent and dependent variables were used. Additionally, the influence of population size was controlled to reduce heteroscedasticity by incorporating mean QALY gains and treatment costs into the regression model. The model is represented as follows:

1 $$\ln \,\,(H_{i} ) = \beta_{1} \ln \,\,\,(E_{i} ) + \beta_{2} X_{i} + \varepsilon i$$

*i*: sample *i*, composed of a drug and its indication of NSCLC in NRDL

H_i_: mean QALY gain of indication *i.*

E_*i*_: mean treatment cost of indication *i.*

X_*i*_: covariates (sex, age, line of treatment, local or overseas drugs, with/without gene mutation, and year of listed) affecting QALYs of indication *i.*

*ε*_*i*_: disturbance term.

*β*_1_: H_*i*_ increases by β_1_% for every 1% increase in E_*i*_*.*

#### Target population

The study focused on patients with NSCLC who underwent therapy listed in the 2021 National Reimbursement Drug List (NRDL). Additional file 1: Appendix Table S2 provides detailed information on the specific drugs, indications, and corresponding Randomized Controlled Trials (RCTs) included in the analysis. A total of thirteen drugs and nine indications were considered, covering a population of approximately 520,000 patients.

We specifically included drugs from the 2021 National Reimbursement Drug List (NRDL) negotiation catalog, which encompassed newly added and renewed products. We excluded drugs from the routine catalog since their inclusion was not based on pharmacoeconomic evidence. Consequently, the association between cost and quality-adjusted life years (QALYs) for drugs in the routine catalog does not reflect the payer threshold.

#### Model input and data source

*Cost* The treatment cost was divided into two components: the cost during progression-free survival (PFS) and the cost during post-progression survival (PPS), where PPS was calculated as the difference between overall survival (OS) and PFS. The cost during PFS was determined by multiplying the duration of PFS (in months) by the monthly treatment cost. The treatment cost calculation was based on the pricing listed in the medical insurance reimbursement and the recommended dosage and administration instructions provided in the drug label for an adult (weight: 60 kg, body surface area: 1.6 m^2^). Similarly, for the PPS cost, the same calculation was applied assuming that follow-up treatments, mainly salvage chemotherapy or best supportive care (BSC), were consistent for all patients, simplifying the models [39]. The exchange rate used for the 2021 average (6.4512 RMB/$) was obtained on June 15, 2022. Since the drugs listed in the 2021 NRDL were officially negotiated and reimbursed at market prices in 2022, all cost data from previous years sourced from the literature were discounted to the year 2022 using a discount rate of 5%.

*QALY* QALYs = PFS*QoL during PFS + PPS*QoL during PPS. The PFS and PPS data were obtained from the clinical trials conducted for each indication, as listed in Additional file 1: Appendix Table S2, while the QoL data were sourced from cross-sectional surveys reported in the literature [26]. It has been shown that there is a disparity between the median OS and PFS reported in clinical trials and the mean OS and PFS used in pharmacoeconomic analysis [24]. To address this discrepancy, we employed a multiplier factor based on the relationship between the median and mean values in the field of lung cancer [24] to transform the median values used in this study into mean values. However, considering the significant instability of such estimation, we incorporated mean OS and PFS in sensitivity analysis, which can independently vary within a range of plus or minus 20%. At the start of this study, for some indications, the median overall survival (OS) was not reported in the available data. To address this issue, we employed the following approach:

- Initially, we searched the published economic evaluation literature to extract any extrapolated median OS. In cases where no relevant extrapolated median OS data were found, we developed a new model to extrapolate. This modeling approach was chosen based on the criteria of minimum Akaike information criterion (AIC) and Bayesian information criterion (BIC).
- For indications where the OS curve was not reported, we assumed that the median OS would be equal to its reported PFS plus the OS data from follow-up treatments. We obtained the published OS data related to these follow-up chemotherapy treatments (salvage chemotherapy and supportive care) from the literature.

*Utility values* Scenario analysis was conducted to explore the impact of different utility value sources. *Base-case:* Different utility values were applied to PFS and PPS, which were sourced from relevant economic literature for the corresponding indications [26] (refer to Additional file 1: Appendix 1 for details). Studies have indicated that the utility values differ between targeted therapy and combination therapy, as well as across different treatment lines [21, 31]. Therefore, these specific utility values were incorporated into the model as part of the scenario analysis. By considering these different utility values, we aimed to explore the potential impact on the outcomes and provide a more comprehensive understanding of the value of different treatment modalities and treatment lines.

*Covariates *The covariates included in the analysis were sex, age, line of treatment, brand origin (local or overseas), gene mutation status (with or without), and the year of listing for the indication-specific drug. However, socio-economic characteristics were not included as covariates in the analysis due to their minimal impact on quality-adjusted life years (QALYs) as observed in clinical trials.

#### Data analysis

Treatment cost was considered as an endogenous variable due to the mutual causality between treatment costs and QALY gains. To address this endogeneity issue, instrumental variables (IVs) and two-stage least squares (2SLS) analysis were employed, which are commonly used methods.

IV needs to satisfy two conditions to be considered valid: relevance to the explanatory variable and independence from the error term. The relevance to the explanatory variable can be assessed through an overidentification test, where a first-stage F-statistic > 10, the chosen IVs are strong instruments. The independence from the error term, known as the exogeneity of the instruments, requires comparing the number of instruments with the number of endogenous variables. The Sargan-Hansen test is commonly used for overidentification testing, and if the p-value > 0.05, the null hypothesis is accepted, indicating that the instruments are exogenous and valid. The selection of instrumental variables relies on research experience and expertise.

The estimation of the payer threshold was conducted using the following approach, as described in the study by Vallejo-Torres [37].

$${\text{Incremental cost per additional QALY = }}\frac{{\Sigma_{i = 1}^{24} \,\,(1\% *E_{i} *n_{i} )}}{{\Sigma_{i = 24}^{24} (\beta_{1} \% *H_{i} *n_{i} )}}$$

*E*_*i*_ represents the treatment cost for indication* i.*

*H*_*i*_ represents the QALY gain of indication *i.*

*n*_*i*_ represents the population size of indication *i*, Population size was estimated using prevalence rate and kept consistent with published budget impact analysis.

Drugs already listed in the NRDL catalog have both higher and lower incremental cost-effectiveness ratios (ICERs). The shadow price method (traditional cost opportunity method) regards the highest of these ICERs as threshold, while the mean opportunity cost method reflects a mean, stable, and generalized threshold over the long term. Each of these methods has its own advantages and drawbacks. This study exclusively employs the latter method for calculation. When a drug exhibits exceptional added value, it can surpass this mean threshold (those drugs already in the NRDL catalog that exceed this mean threshold). The threshold derived from the combined mean opportunity cost and shadow price methods can be interpreted as dynamically adjusting around the mean threshold within a certain range based on the comprehensive value of the drug.

#### Robustness test

Deterministic and probabilistic sensitive analyses were conducted to assess the robustness of the results using different model inputs. Probabilities were followed a beta distribution, while cost parameters followed a gamma distribution, as suggested by Briggs [5]. OS and PFS were varied within a range of plus or minus 20%, the cost of combination therapy was varied within a range of plus or minus 50%, and the remaining parameters were varied within a range of plus or minus 10% (refer to Table 1 in the Appendix). This approach allowed for the exploration of uncertainty and variability in the model, providing a comprehensive assessment of the results.

**Table 1:** Results of threshold, coefficient, and endogeneity tests of base-case and two scenarios

	Base-case	Scenario 1	Scenario 2
Coefficient of cost	0.611 (*p* < 0.001)	0.638 (*p* < 0.001)	0.595 (*p* < 0.001)
Coefficient of age	− 1.656 (*p* = 0.001)	− 1.678 (*p* < 0.001)	− 1.512 (*p* < 0.001)
Coefficient of 2nd line of treatment	− 0.42 (*p* = 0.012)	− 0.271 (*p* = 0.026)	− 0.464 (*p* < 0.001)
Durbin-Wu-Hausman (*p value*)	0.0162	0.0231	0.0213
Threshold (￥)	150,962	112,324	111,824
Threshold/GDP	1.86	1.39	1.38

### Patient willingness-to-pay

#### Patient inclusion and exclusion

Patients diagnosed with NSCLC were prospectively recruited from the Department of Oncology at Jiangsu Cancer Hospital. All included patients have signed informed consent forms and received a compensation of $15 for their participation. The inclusion criteria for enrollment were as follows:

1. Over 40 years of age
2. Diagnosed with NSCLC
3. Previously received at least one systemic chemotherapy
4. Outpatient or inpatient
5. Patients with cognitive impairment were excluded.

#### Survey process and instrument

The questionnaire design used the payment card approach of the contingent valuation method (CVM). In January 2022, a total of 15 volunteers with professional knowledge from the authors' institute and 15 relatives without professional knowledge volunteered to participate in the study. Each participant was provided with a pre-assigned script containing detailed socio-economic status (SES), disease information, and treatment records. The average interview duration was approximately 1 h. Open-ended questions were used to inquire about the threshold they were willing to pay for the new and old intervention measures. Subsequently, we calculated the upper and lower limits of treatment costs for drugs in the NRDL (using the same method as described in ‘‘Model input and data source’’) Sect. By combining these limits and rounding them, the bidding range was determined. Specific bid values within the bidding range were determined based on the distribution of participant choices, as specified in the appendix. The cognitive assessment results indicated that the questionnaire was easily understood. The results of the interviews with the 30 participants are presented in another unpublished study conducted by our team.

From April to June 2022, a group of real patients with NSCLC were recruited for the study. Three pharmacists from Jiangsu Cancer Hospital underwent two training sessions, each lasting 2 h, to familiarize themselves with the study background and enhance their inquiry skills. Face-to-face interviews were conducted with the respondents, with an average duration of 0.75 h. Responses that had logical errors, as determined by both the investigators and researchers, were considered invalid and excluded from the analysis.

The questionnaire was modified after the first round of survey. It ultimately consisted of three sections: baseline characteristics, a QoL survey, and the CVM section. For the detailed questionnaire, please refer to the Appendix.

**Fig. 1:**
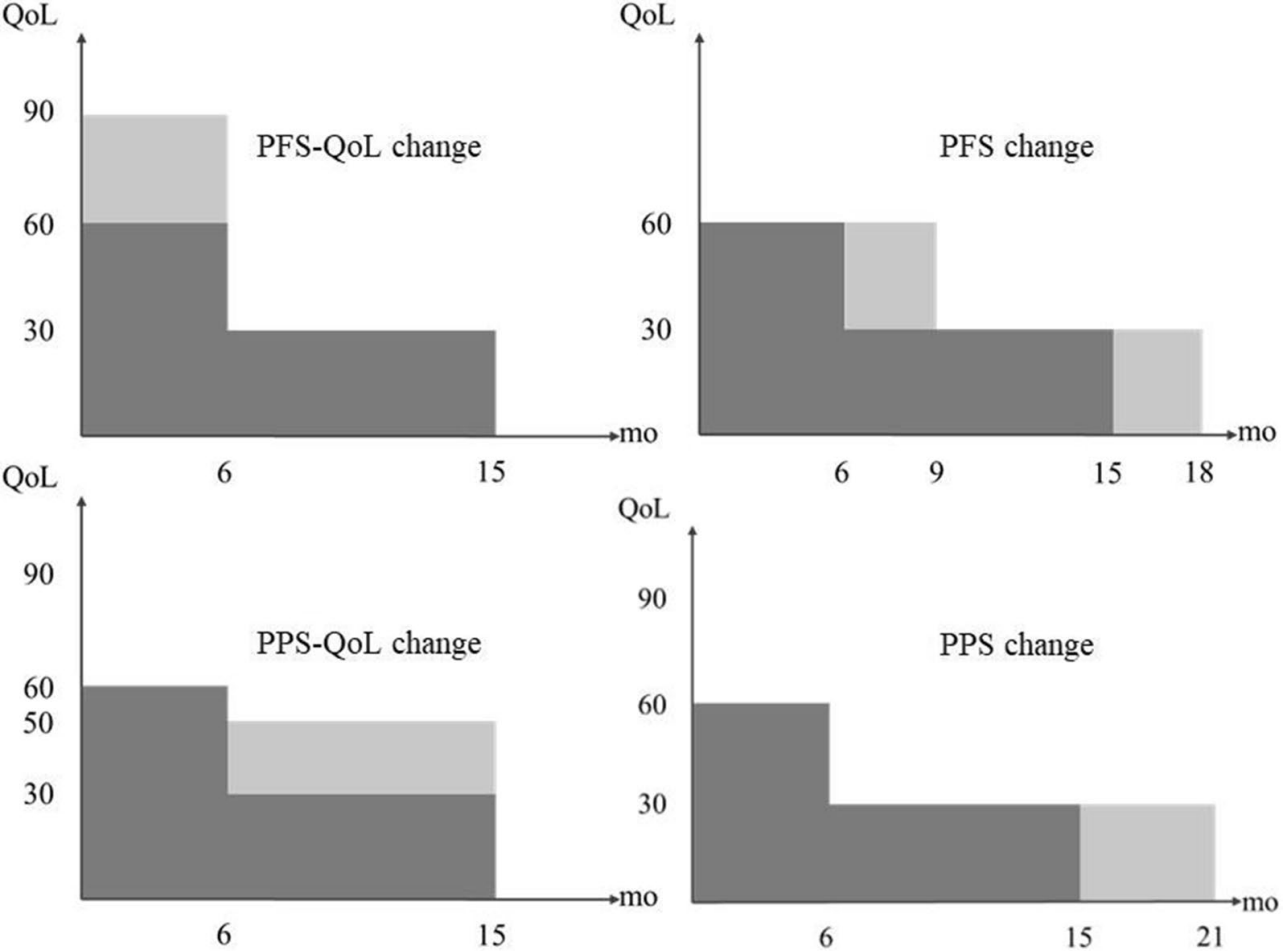
Four types of QALY gains produced by PFS-Qol and PPS improvement

The concept of quality-adjusted life years (QALYs) encompasses four distinct components: PFS, PPS, quality of life during the PFS period (PFS-QoL), and quality of life during the PPS period (PPS-QoL). When one of these four attributes changes while the other attributes keep constant, four different QALYs are generated. We have designed four questions corresponding to these four QALYs. To isolate the influence of health benefits and allow respondents to express their WTP solely based on attribute preferences, we assumed that the four incremental QALYs are equal. (Refer to Fig. 1). The PFS and PPS data were obtained from clinical trials, consistent with ‘‘Model input and data source’’ Sect. The mean values of the experimental group and control group were used to represent the PFS and PPS values before and after the use of the new drug, respectively. The QoL data, consistent with ‘‘Model input and data source’’ Sect. also originate from clinical trials and was rounded to the nearest whole number to represent QoL during the PFS and PPS phases for patients.

It was assumed that respondents would experience improvements survival benefits if they were to receive the new treatment option. They are then asked whether they are willing to pay a certain amount of money for these improvements. If the answer was ‘‘yes,’’ the respondents are asked to choose their maximum WTP from a series of bidding options. If the maximum WTP exceeds the predetermined boundaries of the bidding options, an open-ended question was used to obtain the maximum WTP value from the patient. The WTP values for each unit of incremental QALY are calculated based on each patient's maximum WTP value and the obtained incremental QALY.

#### Data analysis

The formula was used to estimate the WTP of each respondent according to their answers [25], and then the average value was calculated. The formula is shown as follows:

$${{{\text{WTP}}} \mathord{\left/ {\vphantom {{{\text{WTP}}} {{\text{QALY}}}}} \right. \kern-0pt} {{\text{QALY}}}} = {{\left( {\frac{{{\text{WTP}}_{{1}} }}{{{\text{QALY}}_{{1}} }}{ + }\frac{{{\text{WTP}}_{{2}} }}{{{\text{QALY}}_{{2}} }}{ + }...{ + }\frac{{{\text{WTP}}_{{\text{n}}} }}{{{\text{QALY}}_{{\text{n}}} }}} \right)} \mathord{\left/ {\vphantom {{\left( {\frac{{{\text{WTP}}_{{1}} }}{{{\text{QALY}}_{{1}} }}{ + }\frac{{{\text{WTP}}_{{2}} }}{{{\text{QALY}}_{{2}} }}{ + }...{ + }\frac{{{\text{WTP}}_{{\text{n}}} }}{{{\text{QALY}}_{{\text{n}}} }}} \right)} n}} \right. \kern-0pt} n}$$

Each participant (n) was presented with the same set of QALY values, based on real clinical scenarios, ensuring consistency across respondents. As the survival time for advanced lung cancer was relatively short, no discounting was applied to the QALY values.

Differences between preferences toward four types of QALY gains were tested by nonparametric tests. Regression model was conducted to discover the influence of SES on WTP (see below).

Patient WTP = β_1_X_1_ + β_2_X_2_ + β_3_X_3_ + β_4_X_4_ + β_5_X_5_ + β_6_X_6_ + β_7_X_7_ + β_8_X_8_ + ε_i_(X_1_: Age of each patient; X_2_: Sex; X_3_: Disposable income for treatment; X_4_: QoL; X_5_: Whether the patient stopped working early due to disease; X_6_: Education; X_7_: Adverse events; X_8_: Monetary resources).

#### Robustness test

A regression equation was established between WTP and SES based on the existing samples. According to the distribution patterns of various SES in the existing samples, each SES was randomly selected 10,000 times from its respective distribution, forming a sample of 10,000 individuals. The sample data was then plugged into the regression equation to calculate the WTP values. All analyses were conducted in EViews 12 Student Version Lite, R 4.2.1 and Excel 365.

## Results

### The payer threshold

#### Model input

A total of 19 samples were included in the regression equation calculation. In the base-case scenario, the QALY gain was lower compared to scenarios 1 and 2, primarily due to poor QoL during PPS. The mean values of OS and PFS were higher than the median values. The treatment cost ranged from 65,620 to 809,909 yuan.

As of August 11, 2022, among the 19 indications, there are 6 indications for which the median overall survival (OS) has not been reported. The extrapolated OS for Alectinib and Tislelizumab in 1L EGFR negative and ALK negative advanced nonsq-NSCLC was obtained from the published cost-effectiveness analysis literature. The extrapolated OS for Sintilimab in 1L advanced sq-NSCLC was calculated based on the published OS curve and relevant parameters. For Tislelizumab in 1L advanced sq-NSCLC, Ensartinib, and Furmonertinib, the median OS was calculated by adding the median OS of subsequent treatments to their respective PFS (refer to ‘‘Model input and data source’’ Sect for more details). The extrapolated OS for Sintilimab was conducted in R.4.2.1. The lognormal function was applied to extrapolate the OS survival curve and obtain the median OS. The calculation results of QALY and costs for each indication can be found in Additional file 1: Appendix Table 2.

**Table 2:** Baseline characteristics of patients

Number	84
Average age	61.3
Age range	41–84
Male (%)	58 (69.0)
Married (%)	83 (99)
Education (%)	
Primary school and below	20 (23.8)
Junior high school	39 (46.4)
High school/technical secondary school	11 (13.1)
Junior college/university or above	14 (16.7)
Whether to stop working early due to illness (%)	
Yes	43 (51.2)
No	41 (48.8)
Disposable income for illness(RMB)	
In debt	21 (25.0)
0–30,000	30 (35.7)
30,000–90,000	7 (8.3)
90,000–150,000	12 (28.6)
150,000–210,000	5 (6.0)
210,000–300,000	4 (4.8)
300,000–600,000	2 (2.4)
More than 600,000	3 (3.6)
The main source of medical expenditure (%)	
Personal expenditure	51 (60.7)
Assistance from others	33 (39.3)
Diagnosis of lung cancer (%)	
Unknown	4 (4.8)
III	14 (16.7)
IV	66 (78.6)
Duration of disease (%)	
4–7 years	7 (8.3)
3 years	8 (9.5)
2 years	11 (13.1)
1 year	23 (27.4)
< 1 year	32 (38.1)
Survival preference (%)	
QoL increase at PPS stage	11 (13.1)
PPS extension	9 (10.7)
QoL increase at PFS stage	16 (19.1)
PFS extension	48 (57.1)

#### Threshold calculation

In this study, the presence of gene mutation was identified as an instrumental variable after considering factors that could potentially affect treatment costs. The Hausman endogeneity test conducted in the base-case and two scenarios showed evidence of endogeneity. The Cragg-Donald F-statistic value was 12.4553, exceeding the critical values of 15% (based on Stock-Yogo), indicating that the presence of gene mutation was not a weak instrumental variable.

Age (*p* < 0.05) and line of treatment (*p* < 0.05) were retained in models with significant effects (see Table 1). The coefficient of treatment cost indicated that a 1% increase in treatment cost would yield a 0.611% increase in QALY gain, from which the payer threshold in base-case was 150,962 yuan (≈1.86 times the GDP per capita). The results of the two scenario analyses exhibit striking similarity (112,324 and 111,824 yuan, 1.39 and 1.38 per capita GDP), yet both significantly diverge from the baseline analysis. This indicates that the outcomes are markedly influenced by QoL during PFS and PPS, rather than treatment combination or not or treatment lines.

### Patient willingness-to-pay

A total of 85 patients were interviewed, and 84 questionnaires were ultimately included in the statistical analysis. The average age was 61.3 years, and 69% were male. Other baseline characteristics of the respondents are described in Table 2.

#### Response

The response rates for the four types of QALY gains, including PFS, PPS, PFS-QoL, and PPS-QoL, were 86.9%, 85.7%, 83.3%, and 81.0%, respectively. There were 6 individuals who indicated that they were not willing to pay for any type of QALY gain. All of these individuals had an annual income of less than 60,000 yuan, and 5 of them also received financial assistance from others.

#### Patient preference and WTP toward QALY types

The distribution of WTP among respondents was relatively discrete. Due to small samples, the Shapiro‒Wilk test showed that the results of the four QALY gains did not conform to the normal distribution. Consequently, there was no significant difference detected among QALY type-based WTPs (all ***P***s > 0.05). To be more specific, 21 respondents preferred the QALY gains in PPS or PPS-QOL, while 17 respondents preferred that from PFS extension or PFS-QOL improvement (*p* = 0.522). In addition, the preference of QALY gain resulted from prolonged PFS and PPS was like that from QoL in PFS and PPS (*p* = 0.953). (see Table 3).

**Table 3:** The mean and median patient WTP

	PFS WTP	PFS-QoL WTP	PPS WTP	PPS-QoL WTP	WTP for ‘standard QALY’
Average value	157,443	147,991	176,381	158,137	168,376
WTP/GDP	1.94	1.83	2.18	1.95	2.08
Average after trimming 5%	128,103	133,949	144,731	143,871	148,443
WTP/GDP	1.58	1.65	1.79	1.78	1.83
Median value	66,667	66,667	86,667	80,000	106,667
WTP/GDP	0.82	0.82	1.07	0.99	1.32
Preference by Nonparametric tests	PFS WTP				
	0.859	PFS-QoL WTP			
	0.31	0.316	PPS WTP		
	0.392	0.945	0.592	PPS-QoL WTP	
Preference by Nonparametric tests	PFS vs PPS		Life prolong vs QoL increase		
	0.522		0.953		

Since the preferences for the four types of QALY gains were the same, the WTP of the four kinds of QALY gains were incorporated into the WTP for a ‘standard QALY’ by simply adding up, namely:

$$\text{WTP} /\text{QALY} = (\text{PFS}\_\text{WTP} + \text{PFS} - \text{QOL}\_\text{WTP}+\text{PPS}\_\text{WTP} + \text{PPS} - \text{QOL}\_\text{WTP}) / \text{the sum of the QALY gains}$$

The average WTP of each incremental ‘standard QALY’ (after trimming the 5% extreme value) was 148,443 yuan (≈1.83 times the GDP per capita, 95% CI [120,994–175,893], and the median value was 106,667 yuan (≈1.32 times the GDP per capita) (see Table 3).

#### Influencing factors

Linear regression analysis was performed to examine the factors influencing WTP, and the results for both the total population and subgroup analysis can be found in Table 3 in the Appendix. In the total population, several factors were found to have a significant impact on WTP, including age, sex, early cessation of work due to cancer, and disposable income for medical treatment.

Interestingly, the results showed that older individuals had higher WTPs, contrary to what might be expected. Additionally, women showed higher WTPs compared to men. Those who had stopped working early due to cancer showed higher WTPs than those who had not.

Regarding disposable income, respondents with positive income for medical treatment had significantly increased WTPs compared to those with debt status. Furthermore, significant differences were observed between respondents with disposable incomes below 30,000 yuan and those above 30,000 yuan.

In the subgroup analysis, it was found that age did not significantly affect WTP in the female subgroup, unlike in the overall population. A higher level of education was associated with higher WTPs in the female subgroup. On the other hand, in the male subgroup, early cessation of work due to cancer had a significant effect on WTP, which differed from the overall population.

These findings provide insights into the various factors influencing WTP and highlight the importance of considering subgroup differences in understanding individuals' WTP for QALY gains.

### Robustness

**Fig. 2:**
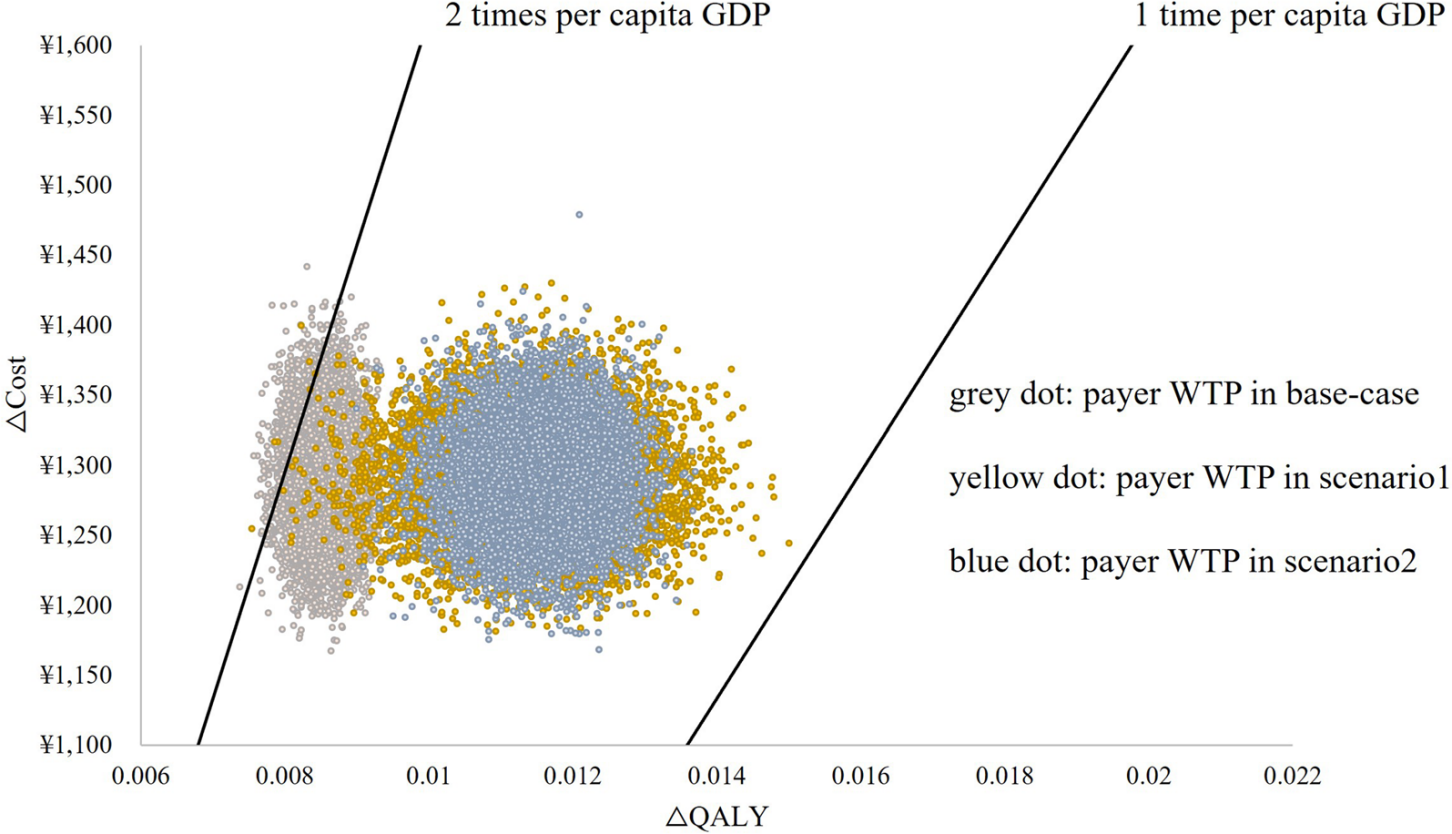
Probabilistic sensitivity analysis of thresholds of base-case and two scenarios

The deterministic sensitivity analysis revealed that the QoL during PFS had the most significant impact on the results, followed by the cost of salvage chemotherapy. The results of the probabilistic sensitivity analysis are presented in Fig. 2. The base-case scenario demonstrated greater stability compared to scenarios 1 and 2, and there was a noticeable gap between the base-case and the other scenarios. This further confirmed that the utility value had a substantial influence on the results.

Moreover, 98.5% of the data points in all three scenarios fell within the range of 1 to 2 times the GDP per capita, showing the robustness of the base-case results.

**Fig. 3:**
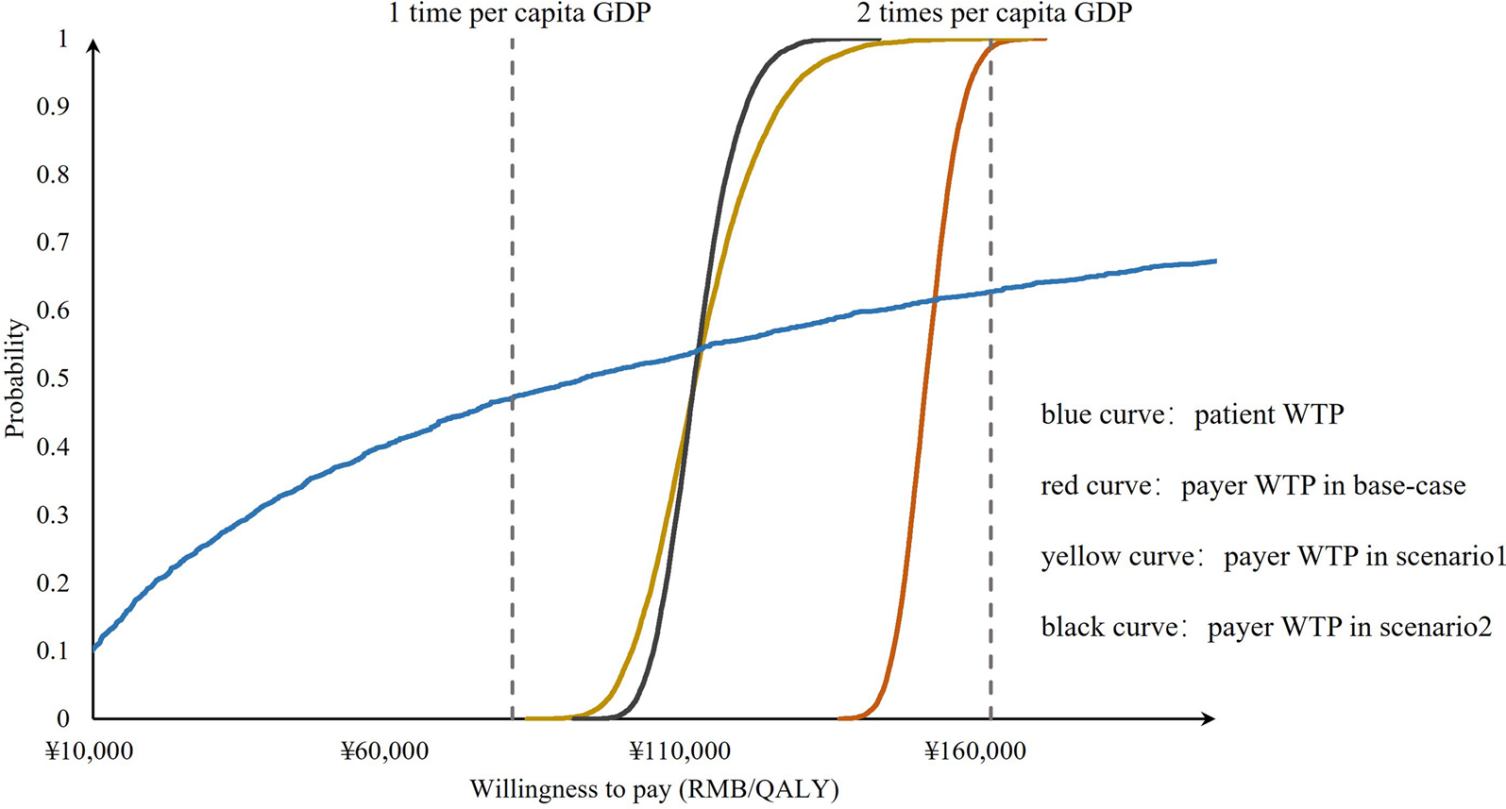
Cumulative probability of willingness to pay from the enlarged sample compared to the payer willingness to pay

The average WTP of the 10,000 resampled respondents was estimated using the regression coefficients described in ‘‘Influencing factors’’, Sect, and the probability distribution of the 10,000 random data points was shown in Fig. 3. Patient WTP exhibited greater variability and less robustness compared to the payer threshold. To enhance the robustness of the patient WTP estimates, a larger sample size of patients would be necessary.

These findings emphasize the importance of conducting sensitivity analyses and highlight the need for larger sample sizes to ensure the reliability and generalizability of the results, particularly when estimating patient WTP.

## Discussion

Payer threshold in our study was nearly three times higher than that of the Chinese general population (0.63 times per capita GDP) using the same approach of mean opportunity cost [28], tower over what experts use in real medical insurance negotiation (0.5–1 times per capita GDP) (Liaowang [23], and slightly higher than that of cancer treatment in South Korea (1.4 times per capita GDP) [41]. Since the cost input we used was from real NRDL prices, we reasonably believed that the Chinese government prioritized cancer drugs. Differences in threshold values across diseases are inherently present, yet it is not necessary for Health Technology Assessment (HTA) decisions to delineate disease-specific thresholds. Instead, disease thresholds would be categorized based on the following distinctions: first, by disease characteristics such as life-threatening diseases, chronic diseases, and infectious diseases, and then by further segmenting based on disease burden, economic burden, clinical unmet needs, and other characteristics.

Given the significant degree of data dispersion, it is deemed that the median value better represents patient WTP. Analogous to payer, mean Patient WTP in our study was slightly higher than that of the Chinese general population (1.2 times per capita GDP) [40], Chinese chronic patients ($4700–7400 and $8799–9446, 0.8–1.3 and 1.1–1.2 per capita GDP) [12, 42] and similar to prior Chinese cancer survey (1.39 per capita GDP) [17].

According to literature patient WTP was frequently at the high end of the result distribution, which stems from taxpayers' belief in the overall affordability of national medical insurance and their ignorance of whether the medical insurance fund can withstand it [36]. To date, the countries that have reported payer threshold using mean opportunity cost are Netherlands [2], South Africa [11], Spain [37], Australia [9], China [28], the United Kingdom [7] and Sweden [34]. Except for Netherlands, all these countries have also conducted WTP researches from patient’s perspective. Among them, patient WTP in Spain [29, 37], Australia [9, 33], China [28, 40] and the UK [7, 33] were higher than payer threshold, while only Sweden was the opposite [34, 35]. In this study, payer threshold was higher than patient WTP, which contrasts with most countries.

Comparing with the aforementioned countries, China operates on a patient and medical insurance co-payment system, where patients bear a relatively significant heavy disease and economic burden, resulting in a lower WTP [32]. Our patient WTP aligns with the median patient WTP of a previous study among Chinese cancer patients [17], confirming the negative attitude to WTP. Meanwhile, given humanitarian, sustaining industry interests and the upward adjustment of cancer threshold by many national healthcare systems, payer provide higher threshold for cancer can be explained when feasible within the financial capacity.

The uncertainty of the literature source data in payer threshold and the limited sample size are the main limitations of our study. To compensate for that, we employed uncertainty analyses to address the parameter sensitivity and generated a large random sample to assess the robustness of patient survey. Additionally, when respondents do not have distinguish between types of small QALY gain, the cognitive bias will be exponentially magnified when directly combined into a ‘standard QALY’ [30]. In this study, respondents’ preferences for the four kinds of QALY gains were different from their self-reports, implying that respondents had biases in understanding different QALY attributes and the WTP toward a standard QALY may have bias as well.

## Conclusion

Our study was the first to assess health value perception from both payer and patient. Cancer patients generally have lower WTP due to their heavy burden. Additionally, our study demonstrated that public payers prioritize listing and payment for drugs that treat cancer patients. In China, a country with a copayment system, the government payer threshold was higher than patient WTP, indicating that medical insurance holds significant decision-making authority, thus temporarily negating the need to consider patient WTP.

### Supplementary Information

**Additional file 1: Table S1.** Parameters of deterministic and probabilistic analyses. **Table S2.** Treatment cost, QALY gain, population size, utility and survival data of drugs and indications. **Table S3.** Impact of baseline characteristics on WTP.

## Data Availability

The datasets used and/or analysed during the current study are available from the corresponding author on reasonable request.
