# Supplementary material for: Estimating the cost-effectiveness threshold of advanced non-small cell lung cancer in China using mean opportunity cost and contingent valuation method
Source: Cost Eff Resour Alloc. 2023 Nov 2;21:80. doi: 10.1186/s12962-023-00487-z (PMC10621116; doi:10.1186/s12962-023-00487-z)
Supplement: Supplementary file 1 — Additional file 1: Table S1. Parameters of deterministic and probabilistic analyses. Table S2. Treatment cost, QALY gain, population size, utility and survival data of drugs and indications. Table S3. Impact of baseline characteristics on WTP. [file 12962_2023_487_MOESM1_ESM.docx]

Table 1 Parameters of deterministic and probabilistic analyses

| Parameters | Mean | Upper | Lower | Resource |
| --- | --- | --- | --- | --- |
| *Rate(%)* |  |  |  |  |
| NSCLC | 0.85 | 0.935 | 0.765 | Rosell R, Karachaliou N. Large-scale screening for somatic mutations in lung cancer[J]. Lancet, 2016,387(10026):1354-1356. |
| Advanced | 0.674 | 0.7414 | 0.6066 | Zeng H, Ran X, An L, et al. Disparities in stage at diagnosis for five common cancers in China: a multicentre, hospital-based, observational study[J]. Lancet Public Health, 2021,6(12): e877-e887. |
| I-II stage | 0.326 | 0.3586 | 0.2934 |  |
| Recurrent | 0.318 | 0.3498 | 0.2862 | Zhang Y, Zheng D, Xie J, et al. Development and Validation of Web-Based Nomograms to Precisely Predict Conditional Risk of Site-Specific Recurrence for Patients With Completely Resected Non-small Cell Lung Cancer: A Multiinstitutional Study[J]. Chest, 2018,154(3):501-511. |
| ADC | 0.6 | 0.66 | 0.54 | Wang P, Zou J, Wu J, et al. Clinical profiles and trend analysis of newly diagnosed lung cancer in a tertiary care hospital of East China during 2011-2015[J]. J Thorac Dis, 2017,9(7):1973-1979. |
| SCC | 0.256 | 0.2816 | 0.2304 |  |
| Large cell carcinoma | 0.006 | 0.0066 | 0.0054 |  |
| SCLC | 0.085 | 0.0935 | 0.0765 |  |
| From 1L to 2L | 0.5829 | 0.64119 | 0.52461 | Ying G Z, Chang J S, Cui L S, et al. Third-line therapy in advanced non-small cell lung cancer[J]. J BUON, 2013,18(4):899-907. |
| From 2L to 3L | 0.521 | 0.5731 | 0.4689 |  |
| EGFR+ | 0.6249 | 0.68739 | 0.56241 | Xue X, Asuquo I, Hong L, et al. Catalog of Lung Cancer Gene Mutations Among Chinese Patients[J]. Front Oncol, 2020,10:1251. |
| ALK+ | 0.0243 | 0.02673 | 0.02187 |  |
| T790M+ | 0.66 | 0.726 | 0.594 | Yu H A, Arcila M E, Rekhtman N, et al. Analysis of tumor specimens at the time of acquired resistance to EGFR-TKI therapy in 155 patients with EGFR-mutant lung cancers[J]. Clin Cancer Res, 2013,19(8):2240-2247. |
| ROS1+ | 0.0259 | 0.02849 | 0.02331 | Zhang Q, Wu C, Ding W, et al. Prevalence of ROS1 fusion in Chinese patients with non-small cell lung cancer[J]. Thorac Cancer, 2019,10(1):47-53. |
| Treatment rate | 0.809 | 0.8899 | 0.7281 | Yang L L, Zhang X C, Yang X N, et al. Lung cancer treatment disparities in China: a question in need of an answer[J]. Oncologist, 2014,19(10):1084-1090. |
| No mutation in ADC | 0.278 | 0.3058 | 0.2502 | Meng H, Guo X, Sun D, et al. Genomic Profiling of Driver Gene Mutations in Chinese Patients With Non-Small Cell Lung Cancer[J]. Front Genet, 2019,10:1008. |
| No mutation in SCC | 0.797 | 0.8767 | 0.7173 |  |
| *Cost(RMB)* |  |  |  |  |
| follow-up cost | 59.2 | 65.12 | 53.28 | Wu B, Gu X, Zhang Q, et al. Cost-Effectiveness of Osimertinib in Treating Newly Diagnosed, Advanced EGFR-Mutation-Positive Non-Small Cell Lung Cancer[J]. Oncologist, 2019,24(3):349-357. |
| salvage chemo/cycle | 1669 | 1835.9 | 1502.1 |  |
| surpportive care/cycle | 359 | 394.9 | 323.1 |  |
| Carboplatin/50mg | 98 | 147 | 49 | <https://db.yaozh.com/> |
| Pemetrexed/50mg | 505 | 757.5 | 252.5 |  |
| Cisplatin/50mg | 76 | 114 | 38 |  |
| Gemcitabine/1000mg | 205 | 307.5 | 102.5 |  |
| paclitaxel/30mg | 473 | 709.5 | 236.5 |  |
| *Utility* |  |  |  |  |
| scenario2 QoL of targeted therapy | 0.787 | 0.8657 | 0.7083 | Shen Y, Wu B, Wang X, et al. Health state utilities in patients with advanced non-small-cell lung cancer in China[J]. J Comp Eff Res, 2018,7(5):443-452. |
| scenario2 QoL of combined therapy | 0.678 | 0.7458 | 0.6102 |  |
| scenario3 QoL of 3L | 0.703 | 0.7733 | 0.6327 |  |
| scenario3 QoL of 1L | 0.856 | 0.9416 | 0.7704 |  |
| scenario3 QoL of 2L | 0.768 | 0.8448 | 0.6912 |  |
| PFS-QoL | 0.804 | 0.8844 | 0.7236 | Nafees B, Lloyd A J, Dewilde S, et al. Health state utilities in non-small cell lung cancer: An international study[J]. Asia-Pacific Journal of Clinical Oncology, 2017,13(5):e195-e203. |
| PPS-QoL | 0.321 | 0.3531 | 0.2889 |  |
| *Overal survival and progression-free survival(months)* | | | |  |
| OS 1 | 13.21 | 15.85 | 10.57 |  |
| OS 2 | 54.99 | 65.99 | 43.99 |  |
| OS 3 | 38.15 | 45.79 | 30.52 |  |
| OS 4 | 48.04 | 57.65 | 38.43 |  |
| OS 5 | 30.81 | 36.97 | 24.65 |  |
| OS 6 | 72.57 | 87.09 | 58.06 |  |
| OS 7 | 25.62 | 30.74 | 20.49 |  |
| OS 8 | 43.04 | 51.65 | 34.43 |  |
| OS 9 | 79.79 | 95.75 | 63.83 |  |
| OS 10 | 39.74 | 47.68 | 31.79 |  |
| OS 11 | 34.41 | 41.29 | 27.53 |  |
| OS 12 | 26.29 | 31.55 | 21.03 |  |
| OS 13 | 29.94 | 35.93 | 23.95 |  |
| OS 14 | 21.87 | 26.24 | 17.49 |  |
| OS 15 | 32.11 | 38.53 | 25.68 |  |
| OS 16 | 28.07 | 33.68 | 22.45 |  |
| OS 17 | 48.61 | 58.34 | 38.89 |  |
| OS 18 | 43.47 | 52.16 | 34.77 |  |
| OS 19 | 18.69 | 22.43 | 14.95 |  |
| PFS 1 | 7.30 | 8.76 | 5.84 |  |
| PFS 2 | 26.77 | 32.12 | 21.42 |  |
| PFS 3 | 14.08 | 16.89 | 11.26 |  |
| PFS 4 | 15.52 | 18.62 | 12.42 |  |
| PFS 5 | 10.62 | 12.74 | 8.49 |  |
| PFS 6 | 27.35 | 32.82 | 21.88 |  |
| PFS 7 | 7.73 | 9.28 | 6.19 |  |
| PFS 8 | 17.25 | 20.70 | 13.80 |  |
| PFS 9 | 40.27 | 48.32 | 32.21 |  |
| PFS 10 | 15.81 | 18.97 | 12.65 |  |
| PFS 11 | 12.35 | 14.82 | 9.88 |  |
| PFS 12 | 7.45 | 8.93 | 5.96 |  |
| PFS 13 | 13.50 | 16.20 | 10.80 |  |
| PFS 14 | 10.47 | 12.57 | 8.38 |  |
| PFS 15 | 13.36 | 16.03 | 10.68 |  |
| PFS 16 | 13.36 | 16.03 | 10.68 |  |
| PFS 17 | 20.71 | 24.85 | 16.57 |  |
| PFS 18 | 20.71 | 24.85 | 16.57 |  |
| PFS 19 | 6.15 | 7.38 | 4.92 |  |

The serial numbers after OS and PFS correspond to the order in Table 2.

Table 2 Treatment cost, QALY gain, population size, utility and survival data of drugs and indications

| **Sample** | **Drug** | **Indication** | **Number** | **per capital QALY(base-case)** | **per capital QALY(scenario1)** | **per capital QALY(scenario2)** | **per capital Cost(￥)** | **base-case PFS-QoL** | **base-case PPS-QoL** | **scenario1 QoL** | **scenario2 QoL** | **median OS(mth)** | **median OFS(mth)** | **mean OS(mth)** | **mean PFS(mth)** | **average age** | **Resource** |
| --- | --- | --- | --- | --- | --- | --- | --- | --- | --- | --- | --- | --- | --- | --- | --- | --- | --- |
| 1 | Anlotinib | 3L+ advacned or relapsed NSCLC | 170317 | 0.65 | 0.87 | 0.77 | 65620.2 | 0.80 | 0.32 | 0.787 | 0.703 | 9.50 | 5.40 | 13.21 | 7.30 | 58 | ALTER0303 https://pubmed.ncbi.nlm.nih.gov/30098152/ |
| 2 | Osimertinib | 1L EGFR positive advanced NSCLC | 23621 | 3.19 | 3.61 | 3.92 | 258433.7 | 0.82 | 0.58 | 0.787 | 0.856 | 38.60 | 18.90 | 54.99 | 26.77 | 60 | FLAURA https://pubmed.ncbi.nlm.nih.gov/33544337/ |
| 3 | Osimertinib | 2L EGFR T790M positive advanced NSCLC | 31805 | 2.31 | 2.50 | 2.44 | 171600.5 | 0.77 | 0.70 | 0.787 | 0.768 | 26.80 | 10.10 | 38.15 | 14.08 | 62 | AURA3 https://pubmed.ncbi.nlm.nih.gov/27959700/ |
| 4 | Crizotinib | 1L ALK positive advanced NSCLC | 5327 | 1.91 | 3.15 | 3.43 | 338926.8 | 0.80 | 0.32 | 0.787 | 0.856 | 33.70 | 11.10 | 48.04 | 15.52 | 49 | A8081029 https://cdn.pfizer.com/pfizercom/clinical%20trials/csr%20synopsis/A8081029%20Public%20Disclosure%20Synopsis_Initial_08AUG2016%20.pdf?KMVlPUWWnVkyRs_LY4LN_hQDEYVJsQtN |
| 5 | Crizotinib | 2L ALK positive advanced NSCLC | 1606 | 1.25 | 2.02 | 1.97 | 223899.5 | 0.80 | 0.32 | 0.787 | 0.768 | 21.70 | 7.70 | 30.81 | 10.62 | 50 | A8081007 https://www.ncbi.nlm.nih.gov/pmc/articles/PMC4201002/ |
| 6 | Crizotinib | ROS1 positive advanced NSCLC | 4112 | 3.04 | 4.76 | 5.18 | 550528.8 | 0.80 | 0.32 | 0.787 | 0.856 | 51.40 | 19.30 | 72.57 | 27.35 | 55 | A8081063 https://pubmed.ncbi.nlm.nih.gov/30980071/ |
| 7 | Ceritinib | 2L ALK positive advanced NSCLC | 1606 | 1.00 | 1.68 | 1.64 | 163766.1 | 0.80 | 0.32 | 0.787 | 0.768 | 18.10 | 5.70 | 25.62 | 7.73 | 49 | CLDK378A2109 NCT02040870 https://www.sciencedirect.com/science/article/pii/S0169500220306772 |
| 8 | Almonertinib | 2L EGFR T790M positive advanced NSCLC | 22718 | 1.85 | 2.82 | 2.75 | 281821.8 | 0.80 | 0.32 | 0.787 | 0.768 | 30.20 | 12.30 | 43.04 | 17.25 | 61 | CXHS1900011 https://file.wuxuwang.com/zhuce/ssypfiles/8eeed6bbc9240b74896d761c5dbb0fa1%E4%B8%8A%E5%B8%82%E5%AE%A1%E8%AF%84%E6%8A%A5%E5%91%8A.pdf |
| 9 | Alectinib | 1L ALK positive advanced NSCLC | 3858 | 3.76 | 5.23 | 5.69 | 809909.0 | 0.80 | 0.32 | 0.787 | 0.856 | 56.90 * | 28.27 | 79.79 | 40.27 | 51 | ALEX https://pubmed.ncbi.nlm.nih.gov/32418886/ https://link.springer.com/article/10.1007/s12325-019-00908-7 |
| 10 | Camrelizumab | 1L EGFR negative and ALK negative advanced nonsq-NSCLC | 22166 | 2.16 | 2.25 | 2.83 | 177421.9 | 0.75 | 0.59 | 0.678 | 0.856 | 27.90 | 11.30 | 39.74 | 15.81 | 59 | NCT03134872 CameL https://pubmed.ncbi.nlm.nih.gov/33347829/ |
| 11 | Sintilimab | 1L EGFR negative and ALK negative advanced nonsq-NSCLC | 18619 | 1.86 | 1.94 | 2.45 | 108593.3 | 0.75 | 0.59 | 0.678 | 0.856 | 24.20 | 8.90 | 34.41 | 12.35 | 61 | ORIENT--11(NCT03607539) https://www.jto.org/article/S1556-0864(20)30595-5/fulltext |
| 12 | Sintilimab | 1L advanced sq-NSCLC | 45366 | 1.00 | 1.49 | 1.88 | 87468.7 | 0.80 | 0.32 | 0.678 | 0.856 | 18.57 ** | 5.50 | 26.29 | 7.45 | 64 | ORIENT--12(NCT03629925) https://pubmed.ncbi.nlm.nih.gov/34048947/ |
| 13 | Tislelizumab | 1L EGFR negative and ALK negative advanced nonsq-NSCLC | 16846 | 2.02 | 1.69 | 2.14 | 108448.3 | 0.86 | 0.77 | 0.678 | 0.856 | 21.10 * | 9.70 | 29.94 | 13.50 | 60 | NCT03663205 RATIONALE 304 https://pubmed.ncbi.nlm.nih.gov/34033975/ https://www.frontiersin.org/articles/10.3389/fphar.2022.935581/full#SM1 |
| 14 | Tislelizumab | 1L advanced sq-NSCLC | 45366 | 1.01 | 1.24 | 1.56 | 93752.2 | 0.80 | 0.32 | 0.678 | 0.856 | 15.50 *** | 7.60 | 21.87 | 10.47 | 60 | RATIONALE 307 https://pubmed.ncbi.nlm.nih.gov/33792623/ https://www.jto.org/article/S1556-0864(19)30020-6/fulltext |
| 15 | Ensartinib | 2L ALK positive advanced NSCLC | 1124 | 1.40 | 2.11 | 2.05 | 232329.0 | 0.80 | 0.32 | 0.787 | 0.768 | 22.60 *** | 9.60 | 32.11 | 13.36 | 52 | [NCT03215693 https://pubmed.ncbi.nlm.nih.gov/31628085/ https://www.ncbi.nlm.nih.gov/pmc/articles/PMC8842297/](https://pubmed.ncbi.nlm.nih.gov/31628085/) |
| 16 | Furmonertinib | 2L EGFR T790M positive advanced NSCLC | 19083 | 1.29 | 1.84 | 1.80 | 151415.4 | 0.80 | 0.32 | 0.787 | 0.768 | 19.80 *** | 9.60 | 28.07 | 13.36 | 61 | NCT03452592 https://pubmed.ncbi.nlm.nih.gov/33780662/ https://pubmed.ncbi.nlm.nih.gov/29858027/ |
| 17 | Dacomitinib | 1L EGFR positive advanced NSCLC | 16535 | 2.13 | 3.19 | 3.47 | 165615.6 | 0.80 | 0.32 | 0.787 | 0.856 | 34.10 | 14.70 | 48.61 | 20.71 | 62 | ARCHER 1050 https://pubmed.ncbi.nlm.nih.gov/28958502/ |
| 18 | Icotinib | 1L EGFR positive advanced NSCLC | 51966 | 2.00 | 2.85 | 3.10 | 162508.9 | 0.80 | 0.32 | 0.787 | 0.856 | 30.50 | 11.20 | 43.47 | 20.71 | 56 | NCT01719536(CONVINCE) https://www.annalsofoncology.org/article/S0923-7534(19)34957-9/fulltext |
| 19 | Icotinib | 2L+ advanced NSCLC | 18283 | 0.75 | 1.23 | 1.20 | 70610.9 | 0.80 | 0.32 | 0.787 | 0.768 | 13.30 | 4.60 | 18.69 | 6.15 | 57 | ICOGEN https://pubmed.ncbi.nlm.nih.gov/23948351/ |

Base-case: Utility of PFS and PPS varies, Scenario 1: Utility of targeted therapy and combined therapy varies, Scenario 2: Utility of different lines of treatment varies.

*The median OS is from the CEA literature; ** The median OS is from the result of parameter extrapolation based on the OS curve in an RCT report; *** The median OS was not reported in the RCT report. The median OS is approximately equal to the median PFS plus the median OS of subsequent treatment. The OS and PFS sources are shown in Table 1 in the Appendix.

Table 3 Impact of baseline characteristics on WTP

|  | Total | | Female（n=18） | | Male（n=41) | |
| --- | --- | --- | --- | --- | --- | --- |
| Variables | Coefficient | P value | Coefficient | P value | Coefficient | P value |
| Sex |  |  |  |  |  |  |
| Female | 0.881 | 0.023 | - | - | - | - |
| Male | Reference category | - | - | - | - | - |
| Age | 0.098 | 0.000 | 0.023 | 0.456 | 0.100 | 0.000 |
| QoL | 0.032 | 0.000 | 0.048 | 0.003 | 0.036 | 0.001 |
| Whether the individual stopped working early due to cancer |  |  |  |  |  |  |
| Did not stop working early due to cancer | Reference category | - | Reference category | - | Reference category | - |
| Stopped working early due to cancer | 1.028 | 0.005 | 3.156 | 0.001 | 0.657 | 0.186 |
| Serious AEs |  |  |  |  |  |  |
| No serious adverse reactions | Reference category | - | Reference category | - | Reference category | - |
| Serious adverse reactions | -0.504 | 0.234 | -1.427 | 0.024 | -0.330 | 0.623 |
| Treatment money source |  |  |  |  |  |  |
| Self | Reference category | - | Reference category | - | Reference category | - |
| Money from others | 0.135 | 0.730 | 2.201 | 0.025 | 0.040 | 0.934 |
| Education |  |  |  |  |  |  |
| Primary school | Reference category | - | Reference category | - | Reference category | - |
| Middle school | 0.324 | 0.452 | 4.085 | 0.009 | 0.088 | 0.872 |
| High school | 1.102 | 0.060 | 5.112 | 0.001 | 0.300 | 0.748 |
| More than high school | 0.541 | 0.368 | 2.199 | 0.020 | 0.224 | 0.763 |
| Money for treatment |  |  |  |  |  |  |
| In debt | Reference category | - | Reference category | - | Reference category | - |
| 0-30 thousand yuan | 0.963 | 0.034 | 0.562 | 0.307 | 1.022 | 0.093 |
| 30-90 thousand yuan | 2.714 | 0.000 | - | - | 2.766 | 0.001 |
| 90-150 thousand yuan | 1.962 | 0.001 | 2.833 | 0.005 | 2.712 | 0.006 |
| ＞150 thousand yuan | 2.979 | 0.000 | 4.152 | 0.001 | 2.815 | 0.000 |
| Money for treatment |  |  |  |  |  |  |
| In debt | -0.563 | 0.228 | -0.480 | 0.387 | -0.511 | 0.418 |
| 0-30 thousand yuan | Reference category | - | Reference category | - | Reference category | - |
| 30-90 thousand yuan | 1.951 | 0.008 | - |  | 2.019 | 0.016 |
| 90-150 thousand yuan | 1.122 | 0.039 | 2.034 | 0.082 | 1.796 | 0.031 |
| ＞150 thousand yuan | 2.161 | 0.000 | 3.723 | 0.004 | 1.966 | 0.003 |
| Money for treatment |  |  |  |  |  |  |
| In debt | -1.497 | 0.077 | - | - | -1.802 | 0.050 |
| 0-30 thousand yuan | -1.739 | 0.359 | - | - | -1.056 | 0.212 |
| 30-90 thousand yuan | Reference category | - | - | - | Reference category | - |
| 90-150 thousand yuan | 0.162 | 0.857 | - | - | 0.529 | 0.612 |
| ＞150 thousand yuan | 1.305 | 0.133 | - | - | 0.852 | 0.371 |
| Money for treatment |  |  |  |  |  |  |
| ＜30 thousand yuan | Reference category | - | Reference category | - | Reference category | - |
| ＞30 thousand yuan | 1.997 | 0.000 | 3.271 | 0.001 | 2.099 | 0.000 |

**Questionnaire**

From 2018 to 2021, NRDL comprised 84 anti-tumor medicines and their indications, which can enhance overall survival by 6 to 70 months and quality-adjusted life years (QALYs) by 0.5 to 9. Medical insurance funds have effectively fulfilled the job of strategic buying and have addressed a wide range of clinical demands. However, it is still worthwhile to investigate if the payment of medical insurance for survival benefits corresponds to patient demand. The purpose of this questionnaire is to study patients' preferences and willingness to pay for improvements in life extension and quality of life provided by new lung cancer treatments, as well as to provide evidence for threshold in China.

This questionnaire consists of four parts——baseline characteristics, quality of life, and willingness to pay. Your privacy will not be involved during completing this questionnaire and the results will be kept confidential. There is no conflict of interest in this questionnaire. Please complete the questionnaire truthfully. Thank you for your cooperation!

Investigator No.: 1

Questionnaire number: A01

**Part I Baseline characteristics**

1. Your gender: ○ Male ○ Female

2. Your age: __________________

3. Your marital status

○Married ○Unmarried ○Divorced ○Widowed

4. Your educational level

○ Primary school and below ○ Junior high school ○ Senior high school/Technical secondary school○ Junior College and University and above

1. What industry do you work in? __________

6. Did you stop working early due to illness?

○ Yes No.

7. Your medical insurance (Multiple options available)

□ Self-financed □ Medical insurance for urban workers

□ Other commercial insurance

□ Medical insurance for urban residents (including new rural cooperative medical insurance) □ Publicly funded medical treatment

8. Your current savings plus all the expected income in the next 3 years, then minus deducting the liabilities, daily expenses (excluding the cost of your lung cancer visit), raising children, and supporting the elderly, your remaining disposable amount is about(RMB)

○ in debt ○ 0-15,000 ○ 15,000-30,000 ○ 30,000-60,000

○ 60,000-90,000 ○ 90,000-150,000 ○150,000-210,000

○ 210,000-300,000 ○ 300,000-600,000 ○ More than 600,000

1. Your main source of medical expenditure is

○Personal expenses

○Assistance from others (such as children support, friends and relatives lending money, charity assistance)

10. What was your last diagnosis of lung cancer? [Example: Stage III Non-small Cell Lung Cancer]

___________

11. In which year you were diagnosed with lung cancer?

In ____________

12. Have you ever had any serious adverse events related to treatment? (Such as prolonged hospitalization, limited self-conscious daily activities such as dressing and eating, emergency treatment, or even life-threatening adverse events) [Multiple options available]

□ No

□ Severe leukopenia □ Severe thrombocytopenia

□ Severe neutrophil count decrease

□ Severe febrile neutropenia □ Severe anemia □ Severe pain

□ Severe allergic reaction □ Severe diarrhea

□ Severe nausea and vomiting □ Severe loss of appetite

□ Other _________________

**Part II Quality of Life**

EQ-5D-5L scale was used here.

**Survival benefit introduction**

The survival benefits of cancer can be divided into life extension and the quality of life improvement.

Life extension consists of **progression-free survival (PFS) and post-progressive survival (PPS)**. **PFS** refers to survival time without disease progression (e.g. cancer did not metastasize, tumor size has not changed, etc.) and **PPS** refers to survival time from progression to death (e.g. cancer spread, etc.).

In terms of quality of life (QoL), a score of 100 is used for perfect health and 0 for death. QoL in general population in China is roughly 95, and in patients with advanced lung cancer is roughly 55.

For any given medicine, the survival benefits comprise progression-free survival (PFS) extension, post-progression survival (PPS) extension, quality of life during PFS improvement(PFS-QoL), and quality of life during PPS improvement (PPS-QoL).

- **Have you understood the concept of “survival benefit”?**

○ 1 ○ 2 ○ 3 ○ 4 ○ 5

- **Which kind of survival benefit do you prefer best?**

○ PFS extends with PFS-QoL, PPS, and PPS-QoL remaining

○ PPS extends with PFS-QoL, PFS, and PPS-QoL remaining

○ PFS-QoL increases with PFS, PPS, and PPS-QoL remaining

○ PPS-QoL increases with PFS-QoL, PFS, and PPS remaining

**Part III The willingness to pay survey**

It is assumed that patients with advanced lung cancer **receive conventional chemotherapy will receive PFS extension for 6 months, PFS-QoL increasing of 60, PPS extension of 9 months, PPS-QoL increasing of 30, and the total out-of-pocket spending will be 60,000 RMB**. Next, new drug launch will require you to pay extra for additional survival benefit compared to conventional chemotherapy.

There are four questions. Please answer how much **extra** money you are willing to pay for the new drug according to above paragraph, your income and preference.

1. Please imagine that a new drug will improve your **PFS-QoL scope from 60 to 90**, and without any affect on your PFS, PPS, PPS-QoL score.

(survived 6 months at 60+ survived 9 months at 30 →survived 6 months at **90**+ survived 9 months at 30)

① Are you willing to pay more for this new drug? (＞60,000 yuan)

○ Yes ○No

-Yes: Please tick the maximum value you will pay extra. If your willingness to pay is less than 3,000 or more than 70,000, please write down the specific value in the respective block.

| ≤3,000 | 5,000 | 8,000 | 10,000 | 12,000 | 14,000 | 16,000 | 18,000 |
| --- | --- | --- | --- | --- | --- | --- | --- |
|  |  |  |  |  |  |  |  |
| 20,000 | 25,000 | 30,000 | 35,000 | 40,000 | 50,000 | 60,000 | ≥70,000 |
|  |  |  |  |  |  |  |  |

-No: Why you don't want to pay?

○I. I don not want to increase treatment costs.

○II. The extra benefit of this new drug is not worthy

○III. The extra benefit of this new drug is worthy, but I am more willing to pay for other things.

○IV. Other ___________________

2、Please imagine that a new drug will improve your **PFS from 6 months to 9 months**, and without any affect on your PFS-QoL, PPS, PPS-QoL score.

(survived 6 months at 60+ survived 9 months at 30 →survived **9 months** at 60+ survived 9 months at 30)

Later part is as same as question 1.

3、Please imagine that a new drug will improve your **PPS-QoL from 30 to 50**, and without any affect on your PFS, PPS, PFS-QoL score.

(survived 6 months at 60+ survived 9 months at 30 →survived 6 months at 60+ survived 9 months at **50**)

Later part is as same as question 1.

4、Please imagine that a new drug will improve your **PD from 9 months to 15 months**, and without any affect on your PFS, PPS, PFS-QoL score.

(survived 6 months at 60+ survived 9 months at 30 →survived 6 months at 60+ survived **15 months** at 30)

Later part is as same as question 1.
